# The Impact of Consumer Subsidy on Green Technology Innovations for Vehicles and Environmental Impact

**DOI:** 10.3390/ijerph17207518

**Published:** 2020-10-15

**Authors:** Juan Zhang, Ziyue Wang, Huiju Zhao

**Affiliations:** 1 Business School, Hohai University, Nanjing 211100, China; hhuzhang@hhu.edu.cn (J.Z.); zy.wang@hhu.edu.cn (Z.W.); 2 Office of Informatization Construction Management, Nanjing University of Finance and Economics, Nanjing 210023, China

**Keywords:** consumer subsidy, green production, energy-saving level, consumer surplus

## Abstract

In the pressure of excessive resource consumption and serious environmental pollution, governments provide various consumer subsidies to promote sales of energy-saving vehicles, including the energy-saving fuel vehicle (FV) and the pure electric vehicle (EV) in the automobile industry. Utilizing a Hotelling model, this paper explores two competing firms’ decisions on the selection of green technology innovations for vehicles, namely producing either the energy-saving FV or the pure EV, while the two vehicles are different from each other on not only the energy-saving level but also the consumer’s acceptance. We further explore the impact of the government’s consumer subsidy on the profits, environment, and consumer surplus. We find that the two competing firms’ equilibrium selections of green technology innovations for vehicles change as the variable manufacturing cost of the pure EV varies. In particular, when the variable manufacturing cost of the pure EV is moderate, the firm with a lower technology capacity for improving the energy-saving level of the FV (i.e., firm 2) will produce the pure EV while the other firm (i.e., firm 1) produces the energy-saving FV, and the converse is not true. In this case, the decreasing variable manufacturing cost of the pure EV will benefit firm 2 and make firm 1 lose in a competing context. In particular, both firms would charge lower retail prices as the variable manufacturing cost of the EV decreases. In addition, we find that although the consumer subsidy could reduce the purchasing cost for the consumer and promote both firms to produce higher energy-saving level vehicles, a firm can still reduce its retail price under certain conditions because of the competition between the two firms. Finally, we prove that the consumer subsidy can be always beneficial to the environment, while it may hurt the consumer surplus and the firms’ profits under certain conditions. The results provide suggestions for governments to adopt an appropriate consumer subsidy program from perspectives of the consumer, environment, and economy.

## 1. Introduction

Environmental awareness has grown drastically over the last several decades. As concerns developed, consumer taste and preference for green products became ubiquitous [1,2]. In response to this change, automotive firms began to produce green vehicles, including not only energy-saving fuel vehicles (FV) but also pure electrical vehicles (EV). The energy-saving FV refers to vehicles involving new energies except petrol, such as electricity and hydrogen. For instance, the hybrid electric vehicle is a new energy-saving FV driven by both diesel engine and electric engine. The pure EVs are driven by electricity only. Compared to the energy-saving FV, the pure EV has a higher energy-saving level, which is more environmentally friendly. However, the low endurance of the EV because of the limited public service infrastructures supporting the operation of EVs, such as charging stations, is unfriendly to the majority of consumers, especially in some developing countries [3]. Moreover, technical difficulties of producing EVs are common issues faced by all automotive firms. Specifically, few automotive firms master key technologies for producing the pure EV, such as Tesla and BYD, and the majority of automotive firms have to produce the EVs with little difference in technical quality, referring to the performance and the energy-saving rate of the EV. In terms of the characteristics of the two types of energy-saving vehicles, automotive firms usually need to make a decision on what type of energy-saving vehicles to produce.

In order to protect the environment, governments introduce various policies to support the development of green vehicles, including the consumer subsidy, the R&D subsidy, and the tax preference. Among them, the consumer subsidy is a general government incentive subsidy focusing on subsidizing the consumers who purchase the green vehicles, such as the energy-saving FVs and the EVs. Typically, a consumer purchasing a greener vehicle would receive a higher consumer subsidy. For instance, the government in China provides subsidies up to RMB 30,000 for each plug-in hybrid electric vehicle and up to RMB 55,000 for each battery EV in 2016 [4].

With the increasing competition in the area of FV and the government financial supports, more and more automotive firms choose to produce pure EVs, especially for the emerging automotive firms. Obviously, the firms producing more environment friendly vehicles will receive more green allowances from the government and be more attractive to consumers with environmental concerns [5]. However, some vehicle firms are not willing to give up the advanced technologies of producing the FVs, as well as the conveniences of the FVs for use. They usually choose to produce the energy-saving FVs to consider both the consumer friendly and environment friendly characters of vehicles.

Motivated by these observations, we seek to study the firms’ selection of the energy-saving vehicle design with the government subsidy support in a competing market. To be specific, we try to answer the following questions: (i) what factors would influence the firm’s selection of the energy-saving vehicle design, namely producing an energy-saving FV or a pure EV? (ii) how does a consumer subsidy program influence the firms’ equilibrium decisions, such as pricing and degree of greenness of vehicles, especially in a competing context? (iii) what are the implications of the effects of the consumer subsidy on the environment, the firms’ profits, and the consumer surplus?

To address these issues, we first consider a base model where two automotive firms compete with each other in a market with no subsidy from the government. Each firm chooses to produce an energy-saving vehicle, either the energy-saving FV or the pure EV. Without loss of generality, we assume that all pure EVs have the undifferentiated energy-saving level because the pure EVs are driven by electric energy only, which releases little carbon emission. In addition, the two firms are assumed to possess different levels of technology capacities related to production of the energy-saving levels of the FVs. Therefore, in addition to the firms’ decisions on the vehicle type (i.e., producing either the energy-saving FV or the pure EV), the firms should make the decisions on the energy-saving level of the FV. For either the energy-saving FV or the pure EV, we consider that all additional costs in improving the energy-saving levels of vehicles (i.e., improving fuel economy and reducing pollution) are variable manufacturing costs. They are incurred by additional green devices to install (catalytic converters to reduce pollution, battery, etc.), more material and parts in the vehicles, more expensive materials and parts to use, and more assembly work to make the vehicles [6]. In particular, we assume that pure EVs have the same energy-saving levels that incur the same variable manufacturing costs, while the variable manufacturing costs of energy-saving FVs depend on their energy-saving levels and the firms’ technology capacities. Finally, consumers purchase the vehicles by comparing their preferences on the energy-saving levels of the vehicles, prices, and brands, as well as the government subsidy. In particular, if the government provides a consumer subsidy, the consumer who purchases the green vehicle will receive an allowance increasing with the energy-saving level. Obviously, consumers purchasing the EV will receive more allowances than those who purchase the energy-saving FV.

By employing a Hotelling model, we obtain the equilibrium outcomes of two competing firms without and with the consumer subsidy following Nash game theory. We obtain some interesting results.

Firstly, there are changing Nash equilibriums as the variable manufacturing cost of the pure EV varies. In addition to both firms choosing the same vehicle type, i.e., producing energy-saving FVs or pure EVs, if the two firms choose different vehicle types, the firm with a lower technology capacity of improving the energy-saving level of the FV (i.e., firm 2) would offer the pure EV, and the other firm (i.e., firm 1) offers the energy-saving FV, while the converse is not true. In this case, the decreasing variable manufacturing cost of the pure EV will benefit firm 2 and make firm 1 lose in a competing context. Therefore, the development of the EV technology may hurt some vehicle firms having the advanced technologies used to produce the energy-saving FV. This finding can well explain why Toyota, the leader of firms producing hybrid vehicles, has disclosed its key technologies in order to shorten the gap of technology capacity with other competitors. We also find that both firms would charge lower retail prices as the variable manufacturing cost of the EV decreases. We further derive conditions under which each Nash equilibrium exists. We see that the consumer subsidy can influence not only the conditions but also the two firms’ equilibrium decisions, and eventually influence the environment, profits, and consumer surplus.

Secondly, we prove that consumer subsidy programs could reduce carbon emissions in a competing context in two ways. The first is making both firms more likely to produce pure EVs instead of energy-saving FVs, and the second is encouraging both firms to increase the energy-saving levels of FVs if they decide to produce FVs. These results reveal approaches of the consumer subsidy to improve the environment. In particular, it indicates that although some consumer subsidies are not high enough to promote sales of pure EVs just as the government expects, they could increase energy-saving levels of FVs, and eventually reduce the carbon emissions. In addition, while one may expect that the consumer subsidy can encourage a firm to charge a higher price for the energy-saving FV and EV, we find that the firm producing the less energy-saving vehicle than the other firm will charge a lower price under the consumer subsidy program than that under no subsidy program. This is because the consumer subsidy would enhance the competitive advantages of the firm producing the higher energy-saving vehicle. The firm producing vehicles with the lower energy-saving vehicle would have to reduce its price to retain its potential consumers. This result proves the effectiveness of consumer subsidiary in improving the environment and provides guidance for firms to charge prices for consumers in a competitive context.

Thirdly, we find the conflicting effects of the consumer subsidy on the two firms’ profits because of the competition between them. Specifically, the firm that offers the higher energy-saving vehicle will get benefits while the other firm always gets the loss from the increasing consumer subsidy. In addition, we are surprised to find that the consumer subsidy could hurt the total profits of the two firms if they offer different vehicle types and the consumer subsidy is in a low level; otherwise, the consumer subsidy always increases the total profit of the two firms.

Finally, we find that the consumer subsidy always benefits the consumers if both two firms offer the energy-saving FVs. This is because the consumer subsidy not only improves the energy-saving levels of the FVs but also subsidizes the consumer who purchases the FV directly. However, if the firm with the lower technology capacity decides to offer the EV while the firm with the higher technology capacity offers the FV, the consumer surplus would be harmed by increasing the consumer subsidy under certain conditions, namely the government offering a small subsidy and there is a high degree of inconvenience of the EV to the consumer. This is because the increased consumer subsidy could induce more consumers to purchase the EVs and the large inconvenience of the EV to the consumer makes such a shift harmful for the consumer surplus. This result indicates that the government should take the supporting infrastructure of EVs into account while launching a consumer subsidy program.

The remainder of the paper is organized as follows. In Section 2, we review the related literature, followed by the introduction of model development in Section 3. Then in Section 4, we solve and discuss the Nash equilibrium results with and without the consumer subsidy, as well as analyze the impact of a consumer subsidy program on the profits, environment, and consumer surplus. Further, in Section 5, we summarize our main results with a conclusion. Future research directions are also given in this section.

## 2. Literature Review

This paper is closely related to the literature about green production, government policy, and competition.

### 2.1. Green Production

As consumer environmental awareness and social responsibility rose, lots of researchers began to pay attention to the issues of green production, including green product line design and pricing. Based on the theory of market segmentation, Chen [7] formulates a quality-based model to analyze the customers’ preferences to green and ordinary products and firms’ strategic decisions on the prices and qualities. Dey et al. [8] explore the impact of strategy inventory and procurement strategy on product design with the consideration of both a development-intensive and a marginal-cost intensive green product. Yu et al. [9] focus on a manufacturer’s decision on green product line design and the production quantity under the government subsidy policy. Shen et al. [10] identify the optimal product line design for green and nongreen products in terms of quality differentiation. There are also many studies analyzing the impact of the government policies on manufacturer’s green production [11,12,13,14,15]. For example, Luo et al. [11] investigate the optimal price discount rate and a subsidy ceiling for the subsidy, which could maximize the expected sales of electric vehicles. Murali et al. [12] study the impact of voluntary ecolabels and mandatory environmental regulation on green product development among competing firms. Jung and Feng [13] propose that the government plays a key role in manufacturer’s green production by offering environmental policies.

Note that most literature on green production of vehicles only involves a type of green technology innovation, namely the energy-saving FV [16,17] or the pure EV [18,19,20]. Lou et al. [16] develop a decision-making model to optimize the fuel economy improvement level and the production of internal combustion vehicles. Huang et al. [17] study the hidden costs for fuel-saving technologies in light-duty vehicles. Zhou et al. [18] investigate a firm’s green technology investment for an EV and the pricing decisions under three possible product designs, namely conventional vehicle only, both conventional vehicle and EV, and EV only. Chen et al. [19] demonstrate the impact of subsidy and credit policy on technological development of battery electric vehicles.

Zhu and He [6] propose two types of green products involving two green technology innovations, namely the marginal cost-intensive green product and the development-intensive green product. Their study analyzes decisions on greenness level of products affected by two green technology innovations, which are distinguished by two cost accounting methods. In our paper, we focus on the impact of consumer subsidy intensive to pure EV and observe firms’ selection of two technology innovations, i.e., the energy-saving technology and the pure electric-vehicle technology. In particular, we distinguish two types of green vehicles from not only their energy-saving levels but also the consumer’s acceptance to them depending on their conveniences of use. We consider that the consumer has a lower awareness on the pure EV compared to the energy-saving FV because of the limited infrastructures supporting the operations for the pure EV.

### 2.2. Government Policy

Our work is closely related to the research that considers government policy as an instrument to improve the environment [7,21,22,23]. Cao et al. [21] examine the impacts of cap-and-trade policy and low carbon subsidy policy on the production and carbon emission reduction level of a manufacturer. Hafezalkotob [22] finds that government can orchestrate green supply chains to fulfill environmental objectives under government financial intervention. Nielsen et al. [23] explore two different government incentives, namely incentive policy on per-unit product and that on the total investment in R&D, provided to manufacturers for adopting green technologies to enhance the green level.

Research on consumer subsidy policy is the most related to our work [24,25,26,27,28]. Zhang et al. [24] analyze the economic and environmental impacts of a consumer subsidy policy and find that the consumer subsidy program can always benefit the environment. Shin et al. [25] compare the consumer subsidy and the tax policy through a case study. They find that the consumer subsidy is a greater incentive for improving environmental benefits than the tax policy. Bian et al. [26] examine and compare the consumer and manufacturer subsidy and find that the former yields a lower abatement and a higher consumption quantity, which lead to higher carbon emissions. In the development of sustainable product manufacturing and remanufacturing decisions, Nielsen et al. [27] compare the direct subsidy to consumers, to the manufacturer to stimulate used product collection, and to the manufacturer to improve product quality.

Besides the environmental impacts, a large number of studies also explore the impact of government policy on firms’ profit and social welfare [10,13,29,30,31,32]. Specifically, based on a product quality model, Gouda et al. [29] analyze the impact of government composite regulations on environmental quality and profits of automakers. Yu et al. [30] present a parsimonious model to determine the optimal subsidy program in different settings considering consumer welfare and manufacturer’s profit, finding that government can improve consumer welfare by developing subsidy programs. Myojo and Ohashi [31] explore the effects of consumer subsidies on industry growth and social welfare.

In our paper, the impacts of the consumer subsidy on the environment, the firms’ profits, and the consumer welfare are all taken into account. In particular, we study the impacts of the consumer subsidy in a competing context while the two types of green technology innovation of vehicles are both considered.

### 2.3. Competition of Green Products

Another related body of literature is the research about the competition of green products [12,18,33,34,35,36]. Huang et al. [33] investigate the impact of a government subsidy program on the manufacturers’ profits with the consideration of the two competing manufacturers where one provides a fuel vehicle and the other offers both fuel and electric vehicles. Zhou [34] develops a game model considering the competition between a brown manufacturer and a green manufacturer in a market with both brown and green consumers. He tries to address how the behavior of green consumers influences the optimal pricing decisions, profits, consumer surplus, and environmental performance. Cohen et al. [35] study two firms competing with quality and prices of the green products. Both R&D and consumer subsidy programs are considered and compared by examining their impacts on the environment and the firms’ profits.

Different from this research mentioned above, we consider two automotive firms competing with not only the quality (i.e., the energy-saving level) and prices but also the type of the green technology innovations of the vehicles. Specifically, a firm has to choose a type of green vehicle to produce, such as the energy-saving FV or the pure EV, while competing with the other firm.

### 2.4. Summary

This paper’s position in the literature is summarized in Table 1. It indicates that the contribution of our work includes three points. Firstly, although lots of research has studied green production in the automotive industry, there are few reports that simultaneously consider the energy-saving technology of the FV and the technology for producing the pure electric vehicle. In particular, energy-saving technology of the FV is one way to increase the green level of FV, while the pure electric vehicle technology such as battery development could make an EV drive without fuel consumption. Besides, as most of the previous studies consider one type of vehicle, namely FV or EV, the consumer awareness on EV and FV cannot be distinguished clearly. In our paper, we distinguish FV and EV from not only their energy-saving levels but also the consumer’s acceptance to them depending on their convenience of use. Secondly, we consider the competition between the two firms not only on the energy-saving levels of vehicles, but also on the selection of green technology innovations, namely choosing either the energy-saving technology of FV or the pure EV technology. Note that all the decisions of the firms will interact with each other. Finally, based on the two points above, we measure the impact of consumer subsidy from three perspectives, i.e., profit, environment, and consumer surplus, while the previous research usually considers one or two perspectives.

## 3. Model Development

There are two vehicle firms, labeled by firm 1 and firm 2. Each of the two vehicle firms chooses to produce either an energy-saving fuel vehicle (FV) or an electric vehicle (EV). They compete with each other in the same market. For the energy-saving FV i,i∈{1,2} produced by firm i, we denote its energy-saving level by $e_{i},e_{i}\in (0,1)$. Suppose that driving a traditional FV will generate a basic carbon emission g, an energy-saving FV with the energy-saving level $e_{i}$ will generate carbon emissions $(1-e_{i})g$. As we all know, an EV is more environmentally friendly than a FV because the EV is driven by the electric battery and the running of an electric battery will incur little carbon emission. Without loss of generality, we assume that the energy-saving level of the EV is 1, which means the carbon emissions driving an EV is 0.

Generally, the government provides subsidies for consumers who purchase green vehicles in the automotive industry [11,25,33]. As China’s New Energy Vehicle Subsidy Program and Product Technical Requirements in 2020 show, the subsidy amount for a single vehicle is based on its energy consumption level. Specifically, if the energy-saving level (relative to the basic energy consumption) is less than 10%, the subsidy will be 0.8 times. Moreover, it will be 1.0 time if the energy-saving level is between 10–25%, and 1.1 times if it is more than 25%. Here, we suppose that a consumer receives a subsidy $s(e_{i})$ from the government for each unit of green vehicle with the energy-saving level $e_{i}(i\in \{1,2\})$. Assume $s(e_{i})$ is an increasing function of the energy-saving level of the vehicle, which is true among existing consumer subsidy programs on green products [9,35]. Without loss of generality, we assume that $s(e_{i})=\phi e_{i}$, where ϕ represents the consumer subsidy for per unit of energy-saving level of vehicle. Notice that ϕ=0 means that the government does not provide a consumer subsidy.

We utilize the Hotelling model to depict the consumer’s heterogeneous preference over two firms. The consumers are uniformly distributed on a [0, 1] line, with the two firms at the two ends. Denote x∈[0,1] as the distance from a customer’s location to firm 1, and thus 1−x is the distance between the customer and firm 2. If firm i decides to produce the FV (or EV), it will charge the price $p_{fi}$ (or $p_{ei}$).

By purchasing a FV produced by firm 1 and firm 2, a consumer’s valuations are given by:

(1) $$v_{f1}=V-\beta x-\lambda (1-e_{1})g-p_{f_{1}}+\phi e_{1}$$

(2) $$v_{f_{2}}=V-\beta (1-x)-\lambda (1-e_{2})g-p_{f_{2}}+\phi e_{2},$$

where V is a basic valuation parameter and λ reflects the consumer’s social responsibility. A higher λ implies a higher social responsibility of the consumer. β measures the consumer’s preference to the firm, and a higher β means a larger consumer’s loyalty to the firm or brand.

By purchasing an EV produced by firm 1 and firm 2, a consumer’s valuations are given by:

(3) $$v_{e1}=V-\beta x-p_{e1}-\delta +\phi$$

(4) $$v_{e2}=V-\beta (1-x)-p_{e2}-\delta +\phi ,$$

where δ(δ>0) means the inconvenience of the EV to the consumer. Although the carbon emission of the EV is small, consumers usually evaluate an EV less than an FV due to its feature of battery and few service facilities, which will lead to a long charging time and a short mileage between charges. A higher δ means the EV is more inconvenient to consumers.

We suppose that consumers’ basic production valuation, V, is sufficiently large so that the market is fully covered. This assumption is standard in Hotelling models [37,38], and it enables us to focus on the interesting and realistic scenario where both firms are competing for limited market demand. In the basic model, we also suppose that the total market demand is deterministic and, without loss of generality, normalized to 1.

For either the energy-saving FV or the pure EV, we consider that all additional costs in improving the energy-saving levels of vehicles (i.e., improving fuel economy and reducing pollution) are variable manufacturing costs. They are incurred by additional green devices to install, more material and parts in the vehicles, more expensive material and parts to use, and more assembly work to make the vehicles [6]. In particular, we assume that pure EVs have the same energy-saving levels that incur the same variable manufacturing costs while the variable manufacturing costs of energy-saving FVs depend on their energy-saving levels and the firms’ technology capacities. Following Krishnan and Zhu [39] and Huang et al. [40], we consider the quadratic forms for the variable manufacturing cost of an energy-saving FV, indicating a convex increasing variable manufacturing cost. We identify the unit variable manufacturing cost of an FV in the following functions.

(5) $$c_{fi}=\theta_{i}e_{i}^{2},i\in \{1,2\},$$

where $\theta_{i}$ is the coefficient reflecting the effect of an increase in the energy-saving of the vehicle on the variable manufacturing cost. A larger $\theta_{i}$ implies that it is costlier for firm i to increase the energy-saving level of the FV. Without loss of generality, we assume that $\theta_{1}<\theta_{2}$.

In particular, because technologies for producing the EV are immature for the majority of vehicle firms, the difference in technologies for producing the EVs is much less than that for producing the FVs in the automotive industry. Without loss of generality, we suppose a fixed variable manufacturing cost $c_{e}$ for producing an EV. For simplicity of analysis, we assume the production cost except the variable manufacturing cost as zero.

The timing of events is as follows. First, both firms simultaneously decide to produce either an energy-saving FV or an EV. Then, the firms that choose to produce the energy-saving FV determine the energy-saving levels for the FV. Finally, simultaneously the two firms set prices for the vehicles they produce.

It is straightforward to derive four possible cases based on the two firms’ selections, denoted by the case FF, FE, EF, EE. In the case FF, both firms choose to produce the energy-saving FVs; in the case FE (EF), firm 1 (2) chooses to produce the energy-saving FV, and the other chooses to produce the EV, and in the case EE, both firms choose to produce the EVs. We can derive the demand for firm i producing FV/EV, denoted by $d_{fi}$/$d_{ei}$, for each case:

(i) In case FF,
  (6) $$d_{f1}=((g\lambda +\phi )(e_{1}-e_{2})+\beta -p_{f1}+p_{f2})/(2\beta ),\;d_{f2}=1-((g\lambda +\phi )(e_{1}-e_{2})+\beta -p_{f1}+p_{f2})/(2\beta )$$
(ii) In case FE,
  (7) $$d_{f1}=((g\lambda +\phi )(e_{1}-1)+\beta -p_{f1}+p_{e2})/(2\beta ),\;d_{e2}=1-((g\lambda +\phi )(e_{1}-1)+\beta -p_{f1}+p_{e2})/(2\beta )$$
(iii) In case EF,
  (8) $$d_{e1}=((g\lambda +\phi )(1-e_{2})+\beta -\delta -p_{e1}+p_{f2})/(2\beta ),\;d_{f2}=1-((g\lambda +\phi )(1-e_{2})+\beta -\delta -p_{e1}+p_{f2})/(2\beta );$$
(iv) In case EE,
  (9) $$d_{e1}=(\beta +p_{e2}-p_{e1})/(2\beta ),\;d_{e2}=1-(\beta +p_{e2}-p_{e1})/(2\beta ).$$

Therefore, firm i’s profit function, denoted by $\pi_{i}^{mn},i\in \{1,2\},m,n\in \{E,F\}$, can be expressed as shown in Table 2.

## 4. Nash Equilibrium Results

### 4.1. The Case without Consumer Subsidy

In this section, we consider that the government provides no consumer subsidy for consumers (i.e., ϕ=0). We develop the Nash perfect equilibrium in the following proposition. We use superscript “*” to denote the equilibrium outcomes.

**Proposition** **1.** *Suppose*$\underline{c}<c_{e}<\bar{c}\;$*and*$\;\theta_{2}-\theta_{1}<12\beta \theta_{1}\theta_{2}/(g^{2}\lambda^{2})$*, we have:*

*(i)*  *if*$c_{e}>c_{h}$*, then (F, F) is the unique pure Nash equilibrium (NE) and the equilibrium outcomes are*$\;e_{1}^{*}=g\lambda /(2\theta_{1})$*,*$e_{2}^{*}=g\lambda /(2\theta_{2})$*,*$p_{f1}^{*}=(-g^{2}\lambda^{2}\theta_{1}+4g^{2}\lambda^{2}\theta_{2}+12\beta \theta_{1}\theta_{2})/(12\theta_{1}\theta_{2})$*, and*
  $$p_{f2}^{*}=(-g^{2}\lambda^{2}\theta_{2}+4g^{2}\lambda^{2}\theta_{1}+12\beta \theta_{1}\theta_{2})/(12\theta_{1}\theta_{2});$$
*(ii)*  *if*$c_{l}<c_{e}<c_{h}$*, then (F, E) is the unique pure NE and the equilibrium outcomes are*$e_{1}^{*}=g\lambda /(2\theta_{1})$*,*$p_{f1}^{*}=(g^{2}\lambda^{2}-g\lambda \theta_{1}+3\beta \theta_{1}+c_{e}\theta_{1}+\delta \theta_{1})/(3\theta_{1})$,
  $$p_{e2}^{*}=(-g^{2}\lambda^{2}+4g\lambda \theta_{1}+12\beta \theta_{1}+8c_{e}\theta_{1}-4\delta \theta_{1})/(12\theta_{1});$$
*(iii)*  *if*$c_{e}<c_{l}$*, then (E, E) is the unique pure NE and the equilibrium outcomes are*
  $$p_{e1}^{*}=\beta +c_{e},\;p_{e2}^{*}=\beta +c_{e};$$

*where*$c_{l}=-(g^{2}\lambda^{2}-4g\lambda \theta_{1}+4\delta \theta_{1})/(4\theta_{1})$*,*$c_{h}=-(g^{2}\lambda^{2}-4g\lambda \theta_{2}+4\delta \theta_{2})/(4\theta_{2})$*,*$\underline{c}=-(g^{2}\lambda^{2}-4g\lambda \theta_{2}$$+12\beta \theta_{2}+4\delta \theta_{2})/(4\theta_{2})$*and*$\bar{c}=-(g^{2}\lambda^{2}-4g\lambda \theta_{1}-12\beta \theta_{1}+4\delta \theta_{1})/(4\theta_{1})$.

Proposition 1 shows that the two firms’ production selections follow two thresholds, including a low threshold (i.e., $c_{l}$) and a high threshold (i.e., $c_{h}$). It shows that when the cost of the EV is sufficient high (low), both firms choose to produce the energy-saving FVs (EVs). When the cost of the EV is moderate, the firm with a lower technology capacity will choose to produce the EV, while the firm with a higher technology capacity chooses to produce the FV. It should be noted that (E, F) is never the NE. That is, as the cost of the EV decreases, the firm with the lower technology capacity (i.e., firm 2) for increasing the energy-saving level of the FV would always produce the EV before the firm with the high technology capacity (i.e., firm 1). This is because firm 2 has less competitive advantages than firm 1 if they both produce the FVs. When the cost of the EV is sufficiently small, firm 2 will produce the EV instead of the FV thus lessening the competition, which could improve its profit. Moreover, as the cost of the EV decreases to a sufficiently low level, leading to firm 1 producing the EV, firm 2 would still produce the EV. Therefore, $c_{h}$ and $c_{l}$ can be considered as two thresholds for firm 2 and firm 1, respectively, lower than which the firm 1 and firm 2 will produce the EV instead of the FV.

For simplification of the analysis, we use the superscripts “EF*”, “FE*”, and “EE*” to represent the (F, F), (F, E), and (E, E) NE outcomes when the government provides no consumer subsidy.

**Corollary** **1.** (10) $$(i)\;\partial p_{f1}^{*}/\partial \theta_{1}<0,\;\partial p_{f2}^{*}/\partial \theta_{2}<0,\;\partial c_{l}/\partial \theta_{1}>0,\;\partial c_{l}/\partial \theta_{2}=0,\;\partial c_{h}/\partial \theta_{2}>0,\;\partial c_{h}/\partial \theta_{1}=0;$$

(11) $$\begin{array}{l} (\mathrm{ii})\;\partial p_{f1}^{FF*}/\partial \lambda >0,\;\mathrm{and}\;\partial p_{f2}^{FF*}/\partial \lambda >0\;\;\mathrm{if}\;4\theta_{1}>\theta_{2}\;\mathrm{holds},\;\partial p_{f2}^{FF*}/\partial \lambda <0\;\mathrm{otherwise};\\ \partial p_{f1}^{FE*}/\partial \lambda >0\;\mathrm{and}\;\partial p_{e2}^{FE*}/\partial \lambda >0. \end{array}$$

Corollary 1/(i) shows that if a firm’s technology capacity for improving the energy-saving level of the FV is higher (i.e., as $\theta_{1}$ or $\theta_{2}$ decreases), the firm will increase the price for the FV. This result is counter-intuitive. Usually, a higher technology capacity means a lower variable manufacturing cost, so the firm should charge a lower price for the FV. However, as the firm’s technology capacity increases (i.e., as $\theta_{1}$ or $\theta_{2}$ decreases), the firm is willing to make more environmental effort (i.e., $\partial e_{1}^{*}/\partial \theta_{1}<0$, $\partial e_{2}^{*}/\partial \theta_{2}<0$), which increases the cost for improving the energy-saving level of FV. Thereby, the firm will increase the price for the FV. In addition, the threshold for a firm engaging in producing the EV is independent of the other firm’s technology capacity and decreases in its own technology capacity. This fact implies that a higher technology capacity for improving the technology capacity will reduce the firm’s willingness to produce the EV.

Corollary 1/(ii) shows the impact of consumer’s social responsibility on the price of FV and EV. Specifically, as the consumer’s social responsibility is higher (i.e., as λ increases), firm 1 would charge a higher price for the FV. However, firm 2, having a lower technology capacity, would charge a higher price for the FV as the social responsibility of the consumer rises, only if its technology capacity is sufficiently high (i.e., $\theta_{2}<4\theta_{1}$); otherwise it would charge a lower price for the FV. This is because in a competing market setting where both two firms produce the FVs, firm 2 (that has a lower technology capacity relative to firm 1) would face increasing competitive pressure from firm 1 as the gap of their technology capacities increase. In particular, the consumers’ increasing social responsibility will enhance the competitive pressure on firm 2. Therefore, when firm 2 faces a sufficiently large competitive pressure because of the large gap of the two firms’ technology capacity, firm 2 would charge a lower price for the FV to address the competition in the market as the social responsibility of the consumer increases. Furthermore, if firm 2 chooses to produce the EV, it will charge the price for the EV increasing in the consumers’ social responsibility because the high energy-saving of the EV could reduce the competitive pressure from firm 1′s energy-saving FV as the consumers’ social responsibility increases.

Notice that if the EV is not considered, the NE of the two firms would be (F, F). To further analyze the impact of the emergence of the EV on the two firms, we derive the effect of the cost of the EV on the firms’ profits.

**Corollary** **2.** (12) $$\partial \pi_{1}^{FE*}/\partial c_{e}>0,\;\partial \pi_{2}^{FE*}/\partial c_{e}<0,\;\partial \pi_{1}^{EE*}/\partial c_{e}=0,\;\partial \pi_{2}^{EE*}/\partial c_{e}=0.$$

When the variable manufacturing cost of the EV is moderate so that firm 2 decides to produce the EV and firm 1 produces the FV, firm 1′s profit will increase in $c_{e}$ and firm 2′s profit will decrease in $c_{e}$. These results are intuitive. As the variable manufacturing cost of the EV decreases, (i.e., $c_{e}$ decreases), firm 2 would find it more profitable to produce the EV. Meanwhile, the competitive power of firm 2 relative to firm 1 would be enhanced. Therefore, firm 1 would gain the profit loss from the increase of $c_{e}$. To further compare the firms’ profits under the NE (F, F) and (F, E), we can easily prove that $\pi_{1}^{FE*}<\pi_{1}^{FF*}$ and $\pi_{2}^{FE*}<\pi_{2}^{FF*}$, which means that the firm with the lower technology capacity will benefit from the development of the EV, while the firm with the higher technology capacity will lose from the emergence of the EV when the variable manufacturing cost of the EV is medium. If the variable manufacturing cost of the EV is sufficiently low, leading to both firms producing the EVs, neither of the two firms would dominate the other, thus the two firms get the same profit. In particular, we find that the two firms’ profits are independent of $c_{e}$.

### 4.2. The Case with Consumer Subsidy

In this section, we examine the scenario in which the government provides consumer subsidies for green vehicles. We develop the following propositions and corollaries to examine the impact of the consumer subsidies on the firms’ selections of vehicle type, equilibrium energy-saving levels for the FVs, equilibrium pricing for the FV/EV, total environmental benefits, and the firms’ profits. We use superscript s* to denote the equilibrium outcomes with the consumer subsidies.

**Proposition** **2.** *Under the consumer subsidy program, suppose*${\underline{c}}^{s}<c_{e}<{\bar{c}}^{s}$*and*$\theta_{2}-\theta_{1}<12\beta \theta_{1}\theta_{2}/{\left(g\lambda +\phi \right)}^{2}$*, we have:*

*(i)*  *if*$c_{e}>c_{h}^{s}$*, then (F, F) is the unique pure NE and the equilibrium outcomes are*
  (13) $$\begin{array}{l} e_{1}^{s*}=(g\lambda +\phi )/(2\theta_{1}),\;e_{2}^{s*}=(g\lambda +\phi )/(2\theta_{2}),\\ p_{f1}^{s*}=(4\theta_{2}-\theta_{1})(2g\lambda +\phi )\phi /(12\theta_{1}\theta_{2})+(-g^{2}\lambda^{2}\theta_{1}+4g^{2}\lambda^{2}\theta_{2}+12\beta \theta_{1}\theta_{2})/(12\theta_{1}\theta_{2}),\;\mathrm{and}\\ p_{f2}^{s*}=(4\theta_{1}-\theta_{2})(2g\lambda +\phi )\phi /(12\theta_{1}\theta_{2})+(-g^{2}\lambda^{2}\theta_{2}+4g^{2}\lambda^{2}\theta_{1}+12\beta \theta_{1}\theta_{2})/(12\theta_{1}\theta_{2}); \end{array}$$
*(ii)*  *if*$c_{l}^{s}<c_{e}<c_{h}^{s}$*, then (F, E) is the unique pure NE and the equilibrium outcomes are*
  (14) $$\begin{array}{l} e_{1}^{s*}=(g\lambda +\phi )/(2\theta_{1}),\;p_{f1}^{s*}=\phi (2g\lambda +\phi -\theta_{1})/(3\theta_{1})+(g^{2}\lambda^{2}-g\lambda \theta_{1}+3\beta \theta_{1}+c_{e}\theta_{1}+\delta \theta_{1})/(3\theta_{1}),\;\mathrm{and}\\ p_{e2}^{s*}=\phi (4\theta_{1}-\phi -2g\lambda )/(12\theta_{1})+(-g^{2}\lambda^{2}+4g\lambda \theta_{1}+12\beta \theta_{1}+8c_{e}\theta_{1}-4\delta \theta_{1})/(12\theta_{1}); \end{array}$$
*(iii)*  *if*$c_{e}<c_{l}^{s}$*, then (E, E) is the unique pure NE and the equilibrium outcomes are*
  (15) $$p_{e1}^{s*}=\beta +c_{e},\;\mathrm{and}\;p_{e2}^{s*}=\beta +c_{e};$$

*where*$c_{l}^{s}=-(g^{2}\lambda^{2}-4g\lambda \theta_{1}+4\delta \theta_{1}+\phi (2g\lambda +\phi -4\theta_{1}))/(4\theta_{1})$*,*$c_{h}^{s}=-(g^{2}\lambda^{2}-4g\lambda \theta_{2}+4\delta \theta_{2}+\phi$$\left(2g\lambda +\phi -4\theta_{2}))/(4\theta_{2}\right)$*,*${\underline{c}}^{s}=-(g^{2}\lambda^{2}-4g\lambda \theta_{2}+12\beta \theta_{2}+4\delta \theta_{2}+\phi (2g\lambda +\phi -4\theta_{2}))/(4\theta_{2})$*and*${\bar{c}}^{s}=$$-(g^{2}\lambda^{2}-4g\lambda \theta_{1}-12\beta \theta_{1}+4\delta \theta_{1}+\phi (2g\lambda +\phi -4\theta_{1}))/(4\theta_{1})$.

Proposition 2 shows similar results to that of Proposition 1. When the government offers the consumer subsidy, both firms choose to adopt the same vehicle type, namely offering the energy-saving FVs (if $c_{e}$ is sufficient small) or offering the EVs (if $c_{e}$ is sufficient large). The two firms would adopt the different vehicle type only if $c_{e}$ is medium. In particular, the firm with a lower technology capacity will offer the EV to address the competition.

By comparing Proposition 2 with Proposition 1, we get the results shown in Corollary 3, which indicate the impacts of the consumer subsidy program on the firms’ decisions in a competing setting.

**Corollary** **3.** (16) $$(i)\;c_{l}^{s}>c_{l},c_{h}^{s}>c_{h};$$

(17) $$(\mathrm{ii})\;e_{i}^{s*}>e_{i}^{*},i\in \{1,2\};$$

(18) $$(\mathrm{iii})\;p_{f1}^{EFs*}>p_{f1}^{EF*};p_{f2}^{EFs*}>p_{f2}^{EF*}\;\theta_{2}<4\theta_{1}\;\mathrm{holds},\;\mathrm{verse}\;\mathrm{vice};$$

(19) $$(\mathrm{iv})\;p_{e2}^{FEs*}>p_{e2}^{FE*};p_{f1}^{FEs*}>p_{f1}^{FE*}\;\theta_{1}<\phi +2g\lambda \;\mathrm{holds},\;\mathrm{verse}\;\mathrm{vice}$$

(20) $$(v)\;p_{ei}^{EEs*}>p_{ei}^{EE*},i\in \{1,2\}.$$

Corollary 3/(i) shows that the consumer subsidy increases the two thresholds for the two firms lower than when the two firms would produce the EV instead of producing the energy-saving FV. Specifically, $c_{l}^{s}$ is for firm 1 and $c_{h}^{s}$ is for firm 2. Corollary 3/(ii) shows that with the consumer subsidy, both firms would like to invest more in increasing the energy-saving levels of the FVs if they produce the energy-saving FVs.

**Figure 1:**
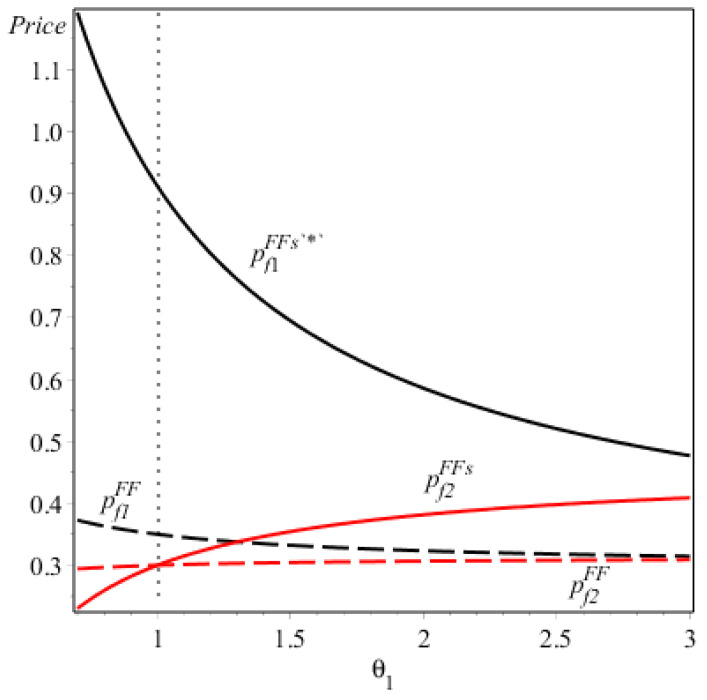
The prices of the two firms under NE (F, F).

Corollary 3/(iii) (see Figure 1) shows the price comparison when both firms offer the energy-saving FVs with and without the consumer subsidy. One may expect that the consumer subsidy would encourage a firm to charge a higher price for the energy-saving FV. This is because the consumer subsidy not only increases the consumers’ valuations on the FV/EV but also increases the energy-saving levels of the FVs. However, Corollary 3/(iii) shows that firm 2 will set a lower price for the energy-saving FV with the consumer subsidy, compared to that without the subsidy if its technology capacity of improving the energy-saving level of the FV is extremely low (i.e., $\theta_{2}>4\theta_{1}$). This is because the amount of the consumer subsidy depends on the energy-saving level of the FV. It is obvious that if both firms offer the FVs, the consumer subsidy would enhance the competitive advantages of the firm with a higher technology capacity (i.e., firm 1) relative to the one with a lower technology capacity (i.e., firm 2). If firm 1 has a sufficiently large competitive advantage compared to firm 2, and the consumer subsidy increases such an advantage, then firm 2 would have to reduce its price for the FV to retain its potential consumers.

**Figure 2:**
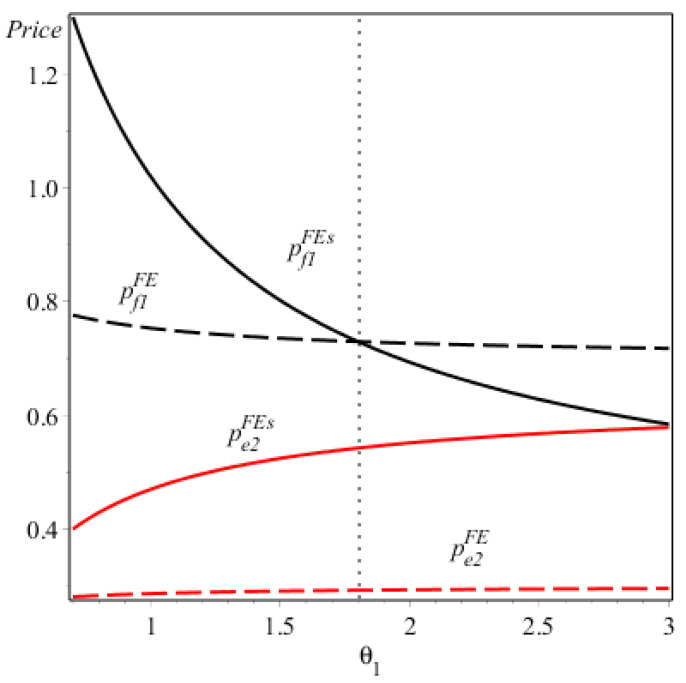
The prices of the two firms under NE (F, E).

Corollary 3/(iv) (see Figure 2) shows the price comparison when firm 1 produces the FV and firm 2 produces the EV in NE. Because the EV is greener than the energy-saving FV, the consumer subsidy would enhance the competitive advantage of firm 2 relative to firm 1. Therefore, firm 2 will charge a higher price for the EV with the consumer subsidy, compared to that without the consumer subsidy. Moreover, instead of reducing the price, firm 1 will charge a higher price for the FV with the consumer subsidy only if its technology capacity is high enough under which it could offer a sufficiently high energy-saving level of FV to retain its potential consumers.

Notice that if the two firms offer the EVs simultaneously, neither would dominate the other. We find that the consumer subsidy makes no effect on the firms’ equilibrium pricing decisions, which is shown by Corollary 3/(v).

## 5. The Impact of Consumer Subsidy

As discussed above, the government’s consumer subsidy program can influence the two firms’ selections of green technology innovations and environmental efforts on improving the energy-saving levels of the FVs, as well as the prices for the vehicles. In this section, we discuss sequentially the impact of the consumer subsidy program on the profit, environment, and consumer surplus. As such, we firstly examine the impact of the consumer subsidy on the profits in Proposition 3.

**Proposition** **3.** (21) $$(i)\;\partial \pi_{1}^{FFs*}/\partial \phi >0,\partial \pi_{2}^{FFs*}/\partial \phi <0,\partial \prod_{}^{FFs*}/\partial \phi >0;$$

(22) $$(\mathrm{ii})\;\partial \pi_{1}^{FEs*}/\partial \phi <0,\partial \pi_{2}^{FEs*}/\partial \phi >0,\;\mathrm{and}\;\mathrm{if}\;0<\phi <\Delta \phi \;\mathrm{holds},\;\partial \prod_{}^{FEs*}/\partial \phi <0,\;\mathrm{otherwise}\;\partial \prod_{}^{FEs*}/\partial \phi >0;$$

(23) $$(\mathrm{iii})\;\partial \pi_{1}^{EEs*}/\partial \phi =0,\partial \pi_{2}^{EEs*}/\partial \phi =0,$$

*where*$\Delta \phi =2\theta_{1}-g\lambda -2\sqrt{\theta_{1}^{2}-c_{e}\theta_{1}-\delta \theta_{1}}$.

Proposition 3 shows the impact of the consumer subsidy coefficient on the profit of firm 1 and 2, and the total channel profit (i.e., the sum of the two firms’ profits, denoted as ∏ under the NE (F, F), (F, E), and (E, E).

**Figure 3:**
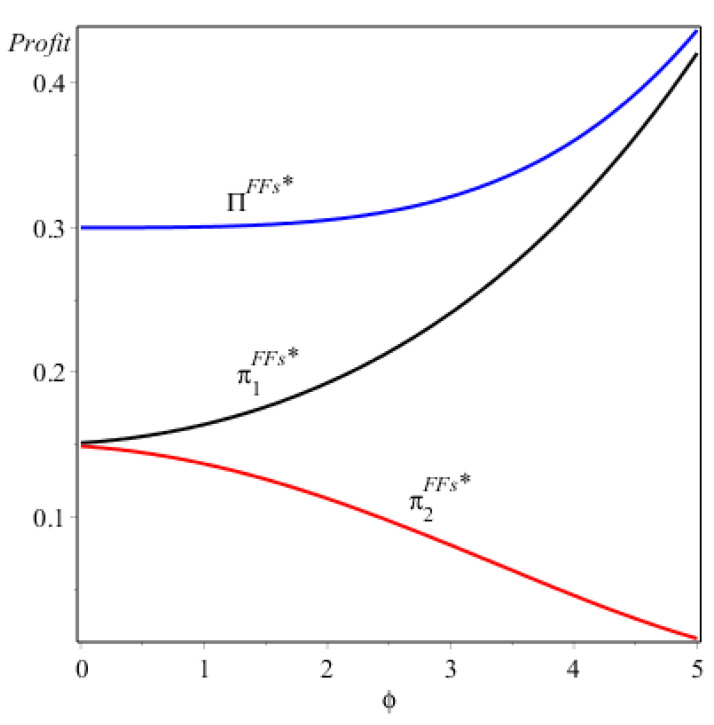
The profits of the two firms under NE (F, F).

As Proposition 3/(i) shows (see Figure 3), when firm 1 and 2 both provide FVs, the profit of firm 1 would increase and that of firm 2 would decrease as the consumer subsidy ϕ increases. The opposite effects of ϕ on the two firms could be attributed to the competition between them. When the two firms both offer energy-saving FVs, firm 1 would offer higher energy-saving levels than firm 2. Obviously, a consumer could get more subsidies from purchasing the energy-saving FV of firm 1 than from purchasing that of firm 2. Therefore, as the consumer subsidy rises, firm 1 would gain more competitive advantages than firm 2, which largely reduces the sales of firm 2. Firm 2 would lose more profits as its sales decrease. In particular, the whole profits (i.e., ∏) are improved under the consumer subsidy program, which implies that the consumer subsidy could take more gains of profits to firm 1 relative to the loss of profits to firm 2 when the two firms both offer the FVs.

**Figure 4:**
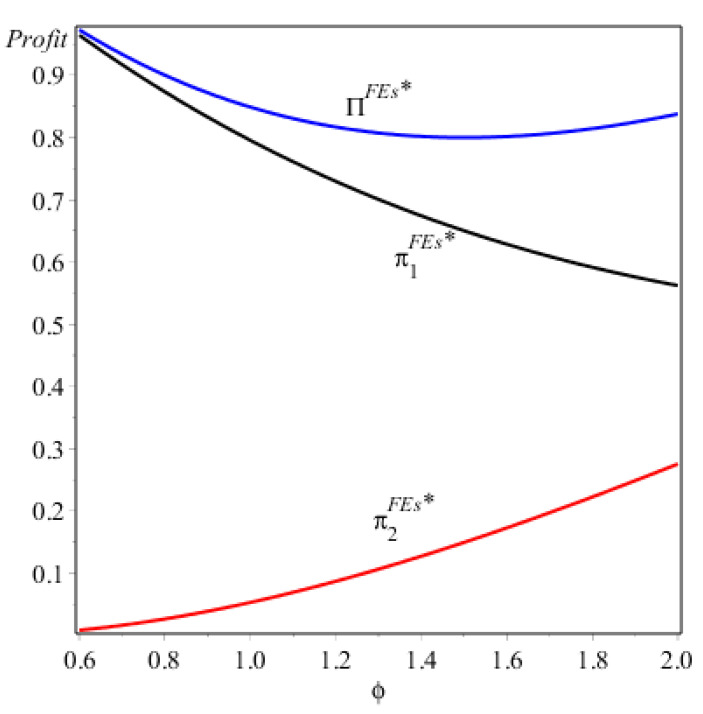
The profits of the two firms under NE (F, E).

As Proposition 3/(ii) (see Figure 4) demonstrates, when firm 1 produces FV and firm 2 provides EV, the consumer subsidy still takes opposite effects on the two firms, but the increasing consumer subsidy will decrease firm 1′s profit and increase firm 2′s profit. This is due to the fact that the EV produced by firm 2 has a higher energy-saving level than the FV produced by firm 1, leading to a higher consumer subsidy for the consumer who chooses the EV. Obviously, an increasing consumer subsidy would reduce the competitive advantages of firm 1 relative to firm 2, which would be harmful for firm 1 and beneficial for firm 2. Furthermore, it should be noted that the whole profits of the two firms might decrease in the consumer subsidy while the intuition is that the government subsidy would be beneficial for the firms. Our result shows that when the government subsidy is sufficiently small (i.e., 0<ϕ<Δϕ), in spite of firm 2′s profit, it could be improved by the increasing the consumer subsidy, firm 1′s profit decreases largely, thus the whole profits of the two firms would be hurt. When firm 1 and 2 both produce EV, as Proposition 3/(iii) shows, we can see that the consumer subsidy has no effect on the two firms’ profits.

In summary, an increasing consumer subsidy cannot be beneficial for both competing firms. In particular, the firm that offers the vehicle with the higher energy-saving level will get benefits from the consumer subsidy. The consumer subsidy could hurt the total profits of the two firms if the two firms offer different vehicle types in NE and the consumer subsidy is at a low level, otherwise the consumer subsidy always increases the total profit of the two firms.

We then define a metric for measuring total environmental benefits. As Shen et al. [10] and Figliozzi [41] indicate, the carbon emissions released by a vehicle can be used to measure the environmental level of the vehicle. Generally, a vehicle releasing more carbon emissions is more harmful to the environment. Note that an energy-saving FV that has an environmental quality $e_{i},(i\in \{1,2\})$ can generate the carbon emissions $(1-e_{i})g$, while an EV generates zero carbon emission. We can write the total carbon emissions of the two firms with the consumer subsidy, denoted by $CE^{mns*},m,n\in \{E,F\}$ under the three NEs in Table 3.

We see that the total carbon emissions are determined by three factors: i) the selection of green technology innovations of the two firms, ii) the demand for vehicles, and iii) the energy-saving levels of the vehicles. By comparing the total carbon emissions with and without the consumer subsidy, we can obtain Proposition 4.

**Proposition** **4.** *Comparing the carbon emissions with and without the consumer subsidy shows that*$CE^{FFs*}<CE^{FF*}$.

Proposition 4 indicates significant effects of the consumer subsidy in terms of protecting the environment. It is generally believed that the consumer subsidy could encourage firms to provide EVs with no carbon emissions and benefit the environment. In particular, when the two firms both produce FVs and no EV is provided, the total carbon emission of all vehicles with the consumer subsidy is still lower than that without the consumer subsidy (i.e., $CE^{FFs*}<CE^{FF*}$). In other words, although the consumer subsidy is originally designed to stimulate the production of EV to reduce the carbon emission, it can still be beneficial to the environment if neither of the firms produce the EV under certain conditions. This is because the energy-saving levels of FVs with the consumer subsidy are higher than that without the subsidy (i.e., $e_{i}^{s*}>e_{i}^{*},i\in \{1,2\}$ which is shown in Corollary 3/(ii)). Therefore, in spite of the consumer subsidy program promoting the two firms’ sales of FVs significantly, the carbon emissions will decline.

Socially responsible firms assess their operational performance from not only profit and environment impact, but also consumer surplus (Shen et al. 2019). In the following, we evaluate the impacts of the consumer subsidy on the consumer surplus, denoted by CS. We first write $CS^{mns*},m,n\in \{E,F\}$ under the three NEs when the government offers the consumer subsidy (Table 4).

By comparing the consumer surpluses with and without the consumer subsidy, we can get Proposition 5.

**Proposition** **5.** (24) $$(i)\;\partial CS^{FFs*}/\partial \phi >0;$$

(25) $$(\mathrm{ii})\;\partial CS^{FEs*}/\partial \phi <0\;\mathrm{if}\;0<\phi <\Delta \phi \;\mathrm{and}\;\delta >\Delta \delta \;\mathrm{both}\;\mathrm{hold},\;\mathrm{and}\;\partial CS^{FEs*}/\partial \phi >0\;\mathrm{otherwise},$$

*where*$\Delta \phi =2\theta_{1}-g\lambda -2\sqrt{\theta_{1}^{2}-c_{e}\theta_{1}-\delta \theta_{1}}$*and*$\Delta \delta =9\beta (2\theta_{1}+g\lambda +\phi )/(2\theta_{1}-g\lambda -\phi )-(4c_{e}\theta_{1}+{\left(g\lambda +\phi \right)}^{2}-$$4(g\lambda +\phi )\theta_{1})/(4\theta_{1})$.

Proposition 5 points out the relationship between the consumer surplus and the consumer subsidy. $\partial CS^{FFs*}/\partial \phi >0$ shows that the consumer subsidy always benefits the consumers if both firms offer the energy-saving FVs. This is because the consumer subsidy not only improves the energy-saving levels of the FVs but also subsidizes the consumer who purchases the FV directly. However, if firm 2 decides to offer the EV while firm 1 offers the FV, the consumer surplus would be reduced as the consumer subsidy increases if the conditions 0<ϕ<Δϕ and δ>Δδ are both satisfied. Specifically, if 0<ϕ<Δϕ holds, indicating that if the government offers a small subsidy, the energy-saving level of firm 1′s FV would be improved slightly. Furthermore, a large δ (i.e., δ>Δδ) means a high degree of inconvenience of the EV to the consumer, leading to a low consumer surplus for the consumers who purchase the EVs. While increasing the consumer subsidy could induce more consumers to purchase the EVs, a large inconvenience of the EV to the consumer makes such a shift harmful for the consumer surplus.

Combining Propositions 3 and 5, we can see that the consumer subsidy offered by the government could hurt the consumer and the firms simultaneously if the subsidy is at a low level and the degree of inconvenience of the EV to consumers is high, when the two competing firms choose the different types of vehicles.

## 6. Conclusions

In order to slow the speed of environmental pollution, it is generally accepted that the government supports the consumer with an incentive subsidy to promote the development of green vehicles in the automobile industry. While the government usually provides a higher subsidy (price discount, etc.) for the consumer to purchase a vehicle with a higher energy-saving level, the pure electric vehicle (EV) would be more attractive to consumers than the energy-saving fuel vehicle (FV) because of its higher energy-saving level. However, the limited supporting infrastructure for the operation of the EV (e.g., charging stations, etc.) reduces the consumer’s valuation on the pure EV. Therefore, the vehicle firms need to decide to produce either the energy-saving FV or the pure EV, especially in a competing context.

This paper constructs an oligopolistic competition game model where two vehicle firms choose to produce the pure EV or the energy-saving FV. The analysis of Nash equilibrium results shows each firm’s optimal green technology innovation selections and price decision for FV or EV, as well as the energy-saving level for the FV (if it decides to produce the FV). Furthermore, while the consumer subsidy supported by the government is taken into account, the impacts of the consumer subsidy on the firms’ profit, environment, and the consumer surplus are examined by comparing the equilibrium outcomes with and without the consumer subsidy.

We find the following results. Firstly, there are changing Nash equilibriums as the variable manufacturing cost of the pure EV varies. In addition to both firms choosing the same vehicle type, i.e., producing energy-saving FVs or pure EVs, if the two firms choose different vehicle types, the firm with a lower technology capacity for improving the energy-saving level of the FV (i.e., firm 2) would offer the pure EV and the other firm (i.e., firm 1) offers the energy-saving FV, while the converse is not true. In this case, the decreasing variable manufacturing cost of the pure EV will benefit firm 2 and make firm 1 lose in a competing context. Therefore, the development of the EV technology may hurt some vehicle firms having the advanced technologies and producing the energy-saving FV. This finding can well explain why Toyota, the leader of firms producing hybrid vehicles, disclosed its key technologies in order to shorten the gap of technology capacity with other competitors. We also find that both firms would charge lower retail prices as the variable manufacturing cost of the EV decreases. We further derive conditions under which each Nash equilibrium exists. We see that the consumer subsidy can influence not only the conditions but also the two firms’ equilibrium decisions, and eventually influence the environment, profits, and consumer surplus.

Secondly, we prove that consumer subsidy programs could reduce carbon emissions in a competing context in two ways. The first is making both firms more likely to produce pure EVs instead of energy-saving FVs, and the second is encouraging both firms to increase energy-saving levels of FVs if they decide to produce FVs. These results reveal approaches of the consumer subsidy to improve the environment. In particular, it indicates that although some consumer subsidies are not high enough to promote sales of pure EVs, just as the government expects, they could increase energy-saving levels of FVs and eventually reduce the carbon emissions. In addition, while one may expect that the consumer subsidy can encourage a firm to charge a higher price for the energy-saving FV and EV, we find that the firm producing the less energy-saving vehicle will charge a lower price under the consumer subsidy program than that under no subsidy program. This is because the consumer subsidy would enhance the competitive advantage of the firm producing the higher energy-saving vehicle. The firm producing vehicles with the lower energy-saving vehicle would have to reduce its price to retain its potential consumers. This result proves the effectiveness of consumer subsidiary in improving the environment and provides guidance for firms to charge prices for consumers in a competitive context.

Thirdly, we find the conflicting effects of the consumer subsidy on the two firms’ profits because of the competition between them. Specifically, the firm that offers the higher energy-saving vehicle will get benefits while the other firm always gets the loss from the increasing consumer subsidy. In addition, we are surprised to find that the consumer subsidy could hurt the total profits of both firms if they offer different vehicle types and the consumer subsidy is at a low level; otherwise, the consumer subsidy always increases the total profit of the two firms.

Finally, we find that the consumer subsidy always benefits the consumers if both two firms offer the energy-saving FVs. This is because the consumer subsidy not only improves the energy-saving levels of the FVs but also subsidizes the consumer who purchases the FV directly. However, if the firm with the lower technology capacity decides to offer the EV while the firm with the higher technology capacity offers the FV, the consumer surplus would be harmed by increasing the consumer subsidy under certain conditions, namely if the government offers a small subsidy and there is a high degree of inconvenience of the EV to the consumer. This is because increasing the consumer subsidy could induce more consumers to purchase the EVs, and the large inconvenience of the EV to the consumer makes such a shift harmful for the consumer surplus. This result indicates that the government should take the supporting infrastructure of EVs into account while launching a consumer subsidy program.

## 7. Future Research

Our research can be further extended in several directions. First, the environmental quality level of EV could be considered in future. In fact, although the percentage of greenhouse gas emissions by electric-driving vehicles is much smaller than that of fuel-driving vehicles, the battery life of EV could also influence the environmental quality indirectly. Second, to be more realistic, we could consider both environmentally and not environmentally conscious consumers in a market. Finally, it is more meaningful to investigate the difference in efficiencies between the consumer subsidy and other policies, such as the manufacturer subsidy, R&D subsidy, and credit policy.

## Figures and Tables

**Table 1:** Positioning of this paper in the literature.

Paper	Green Production	GTI for FV	GTI for EV	Competition	Consumer Subsidy Policy	Other Policy
Zhou [34]	√			√		
Luo et al. [11]	√				√	
Murali et al. [12]	√			√		√
Gouda et al. [29]	√					√
Hadi et al. [14]	√					√
Chen et al. [7]	√					√
Zhang et al. [24]	√					√
Yu et al. [9]	√			√		√
Shen et al. [10]	√					
Zhou et al. [18]	√		√			√
Lou et al. [16]	√	√				√
Chen et al. [19]	√		√			√
Huang et al. [17]	√	√				
Cao et al. [21]	√					√
Hafezalkotob [22]	√			√		√
Yang et al. [36]	√			√		√
Jung and Feng [13]	√			√		√
Shin et al. [25]	√				√	√
Li et al. [32]	√		√	√	√	√
Yu et al. [30]	√			√	√	√
Myojo and Ohashi [31]	√			√	√	
Bian et al. [26]	√		√	√	√	√
Huang et al. [33]	√			√	√	
Zhu and He [6]	√	√	√	√		√
Cohen et al. [35]	√			√	√	√
Our paper	√	√	√	√	√	

Note: GTI refers to green technology innovation.

**Table 2:** The profits of the firms in the four cases.

	Firm 2	F	E
Firm 1			
F		$\pi_{1}^{FF}=(p_{f1}-\theta_{1}e_{1}^{2})d_{f1}$ $\pi_{2}^{FF}=(p_{f2}-\theta_{2}e_{2}^{2})d_{f2}$	$\pi_{1}^{FE}=(p_{f1}-\theta_{1}e_{1}^{2})d_{f1}$ $\pi_{2}^{FE}=(p_{e2}-c_{e})d_{e2}$
E		$\pi_{1}^{EF}=(p_{e1}-c_{e})d_{e1}$ $\pi_{2}^{EF}=(p_{f2}-\theta_{2}e_{2}^{2})d_{f2}$	$\pi_{1}^{EE}=(p_{e1}-c_{e})d_{e1}$ $\pi_{2}^{EE}=(p_{e2}-c_{e})d_{e2}$

Note: E and F means the firm produces the pure electric vehicle (the energy-saving fuel vehicle).

**Table 3:** The total carbon emissions of the two firms in the three NEs.

	Firm 2	F	E
Firm 1			
F		$CE^{EFs*}=(1-e_{1})gd_{f1}+(1-e_{2})gd_{f2}$	$CE^{FEs*}=(1-e_{1})gd_{f1}$
E		/	$CE^{EEs*}=0$

**Table 4:** The consumer surplus under the three NEs.

	Firm 2	F	E
Firm 1			
F		$CS^{FFs*}=\int_{0}^{d_{f1}}v_{f1}dx+\int_{d_{f1}}^{1}v_{f2}dx$	$CS^{FEs*}=\int_{0}^{d_{f1}}v_{f1}dx+\int_{d_{f1}}^{1}v_{e2}dx$
E		-	$CS^{EEs*}=\int_{0}^{d_{e1}}v_{e1}dx+\int_{d_{e1}}^{1}v_{e2}dx$

## Appendix A

**Proof** **of** **Propositions** **1** **and** **2** If a consumer purchases an energy-saving FV produced by firm i,i∈{1,2}, its valuation is given by

(A1) $$v_{f1}=V-\beta x-\lambda (1-e_{1})g-p_{f_{1}}+\phi e_{1}$$

(A2) $$v_{f_{2}}=V-\beta (1-x)-\lambda (1-e_{2})g-p_{f_{2}}+\phi e_{2}$$

If the consumer purchases an EV produced by firm i,i∈{1,2}, a consumer’s valuation is given by

(A3) $$v_{e1}=V-\beta x-p_{e1}-\delta +\phi$$

(A4) $$v_{e2}=V-\beta (1-x)-p_{e2}-\delta +\phi$$

If both firms produce FVs, denoted by case (F, F), we solve $v_{f1}=v_{f_{2}}$ to get $x=((g\lambda +\phi )(e_{1}-e_{2})+\beta -p_{1}+p_{2})/2\beta$ and $1-x=1-((g\lambda +\phi )(e_{1}-e_{2})+\beta -p_{1}+p_{2})/2\beta$, which are the sales of FV by firm 1 and firm 2, respectively. Similarly, the sales under the case (F, E), (E, F), and (E, E) can be derived as shown in Table A1, denoted by$d_{ki}(i\in \{1,2\},k\in \{e,f\})$. Further, the profits of the firms in the four cases can be obtained, denoted by $\pi_{i}^{mn},i\in \{1,2\},m,n\in \{E,F\}$. □

**Table A1:** The sales and profits of the firms in the four cases with a consumer subsidy.

(F, F).	$d_{f1}=((g\lambda +\phi )(e_{1}-e_{2})+\beta -p_{f1}+p_{f2})/(2\beta )$ **;** $d_{f2}=1-((g\lambda +\phi )(e_{1}-e_{2})+\beta -p_{f1}+p_{f2})/(2\beta )$ **;** $\pi_{1}^{FF}=(p_{f1}-\theta_{1}e_{1}^{2})d_{f1}$ **;** $\pi_{2}^{FF}=(p_{f2}-\theta_{2}e_{2}^{2})d_{f2}$ **;**
(F, E)	$d_{f1}=((g\lambda +\phi )(e_{1}-1)+\beta -p_{f1}+p_{e2})/(2\beta )$; $d_{e2}=1-((g\lambda +\phi )(e_{1}-1)+\beta -p_{f1}+p_{e2})/(2\beta )$; $\pi_{1}^{FE}=(p_{f1}-\theta_{1}e_{1}^{2})d_{f1}$; $\pi_{2}^{FE}=(p_{e2}-c_{e})d_{e2}$;
(E, F)	$d_{e1}=((g\lambda +\phi )(1-e_{2})+\beta -\delta -p_{e1}+p_{f2})/(2\beta )$; $d_{f2}=1-((g\lambda +\phi )(1-e_{2})+\beta -\delta -p_{e1}+p_{f2})/(2\beta )$; $\pi_{1}^{EF}=(p_{e1}-c_{e})d_{e1}$;$\pi_{2}^{EF}=(p_{f2}-\theta_{2}e_{2}^{2})d_{f2}$;
(E, E)	$d_{e1}=(\beta +p_{e2}-p_{e1})/(2\beta )$; $d_{e2}=1-(\beta +p_{e2}-p_{e1})/(2\beta )$; $\pi_{1}^{EE}=(p_{e1}-c_{e})d_{e1}$; $\pi_{2}^{EE}=(p_{e2}-c_{e})d_{e2}$.

Note: If the government provides no consumer subsidy, then ϕ=0.

In the case (F, F), we firstly solve $\partial \pi_{1}^{FF}/\partial_{f1}=0$ and $\partial \pi_{2}^{FF}/\partial_{f2}=0$ to get the best response prices of the two firms, namely

(A5) $$p_{f1}=(2\theta_{1}e_{1}^{2}+\theta_{2}e_{2}^{2}+(g\lambda +\phi )(e_{1}-e_{2})+3\beta )/3$$

(A6) $$p_{f2}=(2\theta_{2}e_{2}^{2}+\theta_{1}e_{1}^{2}+(g\lambda +\phi )(e_{2}-e_{1})+3\beta )/3$$

Then, we solve $\partial \pi_{1}^{FF}/\partial_{e1}=0$ and $\partial \pi_{2}^{FF}/\partial_{e2}=0$ to get the optimal energy-saving levels of the two FVs offered by the two firms, namely $e_{1}^{s*}=(g\lambda +\phi )/(2\theta_{1})$ and$e_{2}^{s*}=(g\lambda +\phi )/(2\theta_{2})$. Taking $e_{1}^{s*}$ and $e_{2}^{s*}$ into Equations (A8) and (A9), we can get the equilibrium prices, namely $p_{f1}^{s*}=(4\theta_{2}-\theta_{1})(2g\lambda +\phi )\phi /(12\theta_{1}\theta_{2})+(-g^{2}\lambda^{2}\theta_{1}+4g^{2}\lambda^{2}\theta_{2}+12\beta \theta_{1}\theta_{2})/(12\theta_{1}\theta_{2})$ and $p_{f2}^{s*}=(4\theta_{1}-\theta_{2})(2g\lambda +\phi )\phi /(12\theta_{1}\theta_{2})+(-g^{2}\lambda^{2}\theta_{2}+4g^{2}\lambda^{2}\theta_{1}+12\beta \theta_{1}\theta_{2})/(12\theta_{1}\theta_{2})$.

Similarly, we summarize the equilibrium outcomes about the energy-saving level of FV and the prices under the other cases (Table A2).

**Table A2:** The equilibrium outcomes with a consumer subsidy in the four cases.

(F, F).	$e_{1}^{s*}=(g\lambda +\phi )/(2\theta_{1})$; $e_{2}^{s*}=(g\lambda +\phi )/(2\theta_{2})$; $p_{f1}^{s*}=(4\theta_{2}-\theta_{1})(2g\lambda +\phi )\phi /(12\theta_{1}\theta_{2})+(-g^{2}\lambda^{2}\theta_{1}+4g^{2}\lambda^{2}\theta_{2}+12\beta \theta_{1}\theta_{2})/(12\theta_{1}\theta_{2})$; $p_{f2}^{s*}=(4\theta_{1}-\theta_{2})(2g\lambda +\phi )\phi /(12\theta_{1}\theta_{2})+(-g^{2}\lambda^{2}\theta_{2}+4g^{2}\lambda^{2}\theta_{1}+12\beta \theta_{1}\theta_{2})/(12\theta_{1}\theta_{2})$; $\pi_{1}^{FFs*}={\left(12\beta \theta_{1}\theta_{2}+(\theta_{2}-\theta_{1}){\left(g\lambda +\phi \right)}^{2}\right)}^{2}/(288\beta \theta_{1}^{2}\theta_{2}^{2})$; $\pi_{2}^{FFs*}={\left(12\beta \theta_{1}\theta_{2}+(\theta_{1}-\theta_{2}){\left(g\lambda +\phi \right)}^{2}\right)}^{2}/(288\beta \theta_{1}^{2}\theta_{2}^{2})$;
(F, E)	$e_{1}^{s*}=(g\lambda +\phi )/(2\theta_{1})$; $p_{f1}^{s*}=\phi (2g\lambda +\phi -\theta_{1})/(3\theta_{1})+(g^{2}\lambda^{2}-g\lambda \theta_{1}+3\beta \theta_{1}+c_{e}\theta_{1}+\delta \theta_{1})/(3\theta_{1})$; $p_{e2}^{s*}=\phi (4\theta_{1}-\phi -2g\lambda )/(12\theta_{1})+(-g^{2}\lambda^{2}+4g\lambda \theta_{1}+12\beta \theta_{1}+8c_{e}\theta_{1}-4\delta \theta_{1})/(12\theta_{1})$; $\pi_{1}^{FEs*}={\left((-4g\lambda +12\beta +4c+4\delta -4\phi )\theta_{1}+{\left(g\lambda +\phi \right)}^{2}\right)}^{2}/(288\beta \theta_{1}^{2})$; $\pi_{2}^{FEs*}={\left((4g\lambda +12\beta -4c-4\delta +4\phi )\theta_{1}-{\left(g\lambda +\phi \right)}^{2}\right)}^{2}/(288\beta \theta_{1}^{2})$;
(E, F)	$e_{2}^{s*}=(g\lambda +\phi )/(2\theta_{2})$; $p_{f1}^{s*}=(g\lambda +3\beta +2c-\delta +\phi )/3-{\left(g\lambda +\phi \right)}^{2}/(12\theta_{2})$; $p_{e2}^{s*}=(-g\lambda +3\beta +c+\delta -\phi )/3+{\left(g\lambda +\phi \right)}^{2}/(12\theta_{2})$; $\pi_{1}^{EFs*}={\left((4g\lambda +12\beta -4c-4\delta +4\phi )\theta_{2}-{\left(g\lambda +\phi \right)}^{2}\right)}^{2}/(288\beta \theta_{2}^{2})$; $\pi_{2}^{EFs*}={\left((-4g\lambda +12\beta +4c+4\delta -4\phi )\theta_{2}+{\left(g\lambda +\phi \right)}^{2}\right)}^{2}/(288\beta \theta_{2}^{2})$;
(E, E)	$p_{e1}^{s*}=\beta +c_{e};p_{e2}^{s*}=\beta +c_{e}$; $\pi_{1}^{EEs*}=\beta /2;\pi_{2}^{EEs*}=\beta /2.$

To ensure the prices and sales of vehicles are non-negative, we take $e_{1}^{s*}$, $e_{2}^{s*}$, $p_{f1}^{s*}$, and $p_{e2}^{s*}$ into $d_{f1}$ and $d_{f2}$ under the case (F, F), and then solve $d_{f1}>0$ and $d_{f2}>0$ to get the constraint condition $\theta_{2}-\theta_{1}<12\beta \theta_{1}\theta_{2}/{\left(g\lambda +\phi \right)}^{2}$. Similarly, under the case (F, E) and case (E, F), the conditions $d_{1}>0$ and $d_{2}>0$ should be satisfied. It is easy to get the final constraint conditions on $c_{e}$, namely ${\underline{c}}^{s}<c_{e}<{\bar{c}}^{s}$, where ${\underline{c}}^{s}=-(g^{2}\lambda^{2}-4g\lambda \theta_{2}+12\beta \theta_{2}+4\delta \theta_{2}+\phi (2g\lambda +\phi -4\theta_{2}))/(4\theta_{2})$ and ${\bar{c}}^{s}=-(g^{2}\lambda^{2}-4g\lambda \theta_{1}-12\beta \theta_{1}+4\delta \theta_{1}+\phi (2g\lambda +\phi -4\theta_{1}))/(4\theta_{1})$. Note that the $d_{1}$ and $d_{2}$ under the case (E, E) are always positive.

The game activities by the two firms about the four possible cases are shown in Table A3.

**Table A3:** The equilibrium conditions in the four cases with a consumer subsidy.

	Firm 2	F	E
Firm 1			
F		$\pi_{1}^{FF}>\pi_{1}^{EF}\to c_{1}<c_{e}<c_{2}$ $\pi_{2}^{FF}>\pi_{2}^{FE}\to c_{3}<c_{e}<c_{4}$	$\pi_{1}^{FE}>\pi_{1}^{EE}\to c_{e}<c_{5}$ or $c_{e}>c_{3}$ $\pi_{2}^{FE}>\pi_{2}^{FF}\to c_{e}<c_{1}$ or $c_{e}>c_{2}$
E		$\pi_{1}^{EF}>\pi_{1}^{FF}\to c_{e}<c_{3}$ or $c_{e}>c_{4}$ $\pi_{2}^{EF}>\pi_{2}^{EE}\to c_{e}<c_{6}$ or $c_{e}>c_{1}$	$\pi_{1}^{EE}>\pi_{1}^{FE}\to c_{5}<c_{e}<c_{3}$ $\pi_{2}^{EE}>\pi_{2}^{EF}\to c_{6}<c_{e}<c_{1}$

(i) if the Nash equilibrium case is (F, F), then the conditions $\pi_{1}^{FF}>\pi_{1}^{EF}$ and $\pi_{2}^{FF}>\pi_{2}^{FE}$ should be satisfied. Thus, we get the constraint conditions on $c_{e}$, i.e., $c_{1}<c_{e}<c_{2}$ and $c_{3}<c_{e}<c_{4}$, where $c_{1}=-(g^{2}\lambda^{2}-4g\lambda \theta_{2}+4\delta \theta_{2}+\phi (2g\lambda +\phi -4\theta_{2}))/(4\theta_{2})$, $c_{2}=(g\lambda +6\beta -\delta +\phi )+(\theta_{1}-2\theta_{2}){\left(g\lambda +\phi \right)}^{2}/(4\theta_{1}\theta_{2})$, $c_{3}=-(g^{2}\lambda^{2}-4g\lambda \theta_{1}+4\delta \theta_{1}+\phi (2g\lambda +\phi -4\theta_{1}))/(4\theta_{1})$, $c_{4}=(g\lambda +6\beta -\delta +\phi )+(\theta_{2}-2\theta_{1}){\left(g\lambda +\phi \right)}^{2}/(4\theta_{1}\theta_{2})$.
(ii) if the Nash equilibrium case is (F, E), then the conditions $\pi_{1}^{FE}>\pi_{1}^{EE}$ and $\pi_{2}^{FE}>\pi_{2}^{FF}$ should be satisfied. Thus, we get the constraint conditions on $c_{e}$, i.e., $c_{e}<c_{5}$ or $c_{e}>c_{3}$, and $c_{e}<c_{1}$ or $c_{e}>c_{2}$, where $c_{5}=-(g^{2}\lambda^{2}-4g\lambda \theta_{1}+4\delta \theta_{1}+24\beta \theta_{1}+\phi (2g\lambda +\phi -4\theta_{1}))/(4\theta_{1})$.
(iii) if the Nash equilibrium case is (E, F), then the conditions $\pi_{1}^{EF}>\pi_{1}^{FF}$ and $\pi_{2}^{EF}>\pi_{2}^{EE}$ should be satisfied. Thus, we get the constraint conditions on $c_{e}$ that $c_{e}<c_{3}$ or $c_{e}>c_{4}$, and $c_{e}<c_{6}$, or $c_{e}>c_{1}$, where $c_{6}=-(g^{2}\lambda^{2}-4g\lambda \theta_{2}+4\delta \theta_{2}+24\beta \theta_{2}+\phi (2g\lambda +\phi -4\theta_{2}))/(4\theta_{2})$.
(iv) if the Nash equilibrium case is (E, E), then the conditions $\pi_{1}^{EE}>\pi_{1}^{FE}$ and $\pi_{2}^{EE}>\pi_{2}^{EF}$ should be satisfied. Thus, we get the constraint conditions on $c_{e}$, i.e., $c_{5}<c_{e}<c_{3}$ and $c_{6}<c_{e}<c_{1}$.

Based on the calculation above, we assume that $\theta_{2}-\theta_{1}<12\beta \theta_{1}\theta_{2}/{\left(g\lambda +\phi \right)}^{2}$ and ${\underline{c}}^{s}<c_{e}<{\bar{c}}^{s}$. It is easy to prove that $c_{5}<c_{6}<{\underline{c}}^{s}<c_{3}<c_{1}<{\bar{c}}^{s}<c_{2}<c_{4}$. We denote $c_{3}$ as $c_{l}^{s}$, and $c_{1}$ as $c_{h}^{s}$, where $c_{l}^{s}=-(g^{2}\lambda^{2}-4g\lambda \theta_{1}+4\delta \theta_{1}+\phi (2g\lambda +\phi -4\theta_{1}))/(4\theta_{1})$ and $c_{h}^{s}=-(g^{2}\lambda^{2}-4g\lambda \theta_{2}+4\delta \theta_{2}+\phi (2g\lambda +\phi -4\theta_{2}))/(4\theta_{2})$.

By integrating the constraint conditions of each case, we can deduce: (i) if the Nash equilibrium case is (F, F), the condition $c_{h}^{s}<c_{e}<{\bar{c}}^{s}$ should be satisfied; (ii) if the Nash equilibrium case is (F, E), the condition $c_{l}^{s}<c_{e}<c_{h}^{s}$ should be satisfied; (iii) the case (E, F) would not be the Nash equilibrium; or (iv) if the Nash equilibrium case is (E, E), then the condition ${\underline{c}}^{s}<c_{e}<c_{l}^{s}$ should be satisfied.

The Proposition 2 is proven.

If the government provides no consumer subsidy, then ϕ=0. Thus, the equilibrium outcomes and conditions without a consumer subsidy can be shown (Table A4). For a more detailed proving process, please refer to the proof of Proposition 2.

**Table A4:** The equilibrium outcomes and conditions without a consumer subsidy.

Cases	Conditions	Equilibrium Outcomes
(F, F)	$c_{e}>c_{h}^{}$	$e_{1}^{*}=g\lambda /(2\theta_{1})$; $e_{2}^{*}=g\lambda /(2\theta_{2})$; $p_{f1}^{FF*}=(-g^{2}\lambda^{2}\theta_{1}+4g^{2}\lambda^{2}\theta_{2}+12\beta \theta_{1}\theta_{2})/(12\theta_{1}\theta_{2})$; $p_{f2}^{FF*}=(-g^{2}\lambda^{2}\theta_{2}+4g^{2}\lambda^{2}\theta_{1}+12\beta \theta_{1}\theta_{2})/(12\theta_{1}\theta_{2})$; $\pi_{1}^{FF*}={\left(12\beta \theta_{1}\theta_{2}+(\theta_{2}-\theta_{1}){\left(g\lambda +\phi \right)}^{2}\right)}^{2}/(288\beta \theta_{1}^{2}\theta_{2}^{2})$; $\pi_{2}^{FF*}={\left(12\beta \theta_{1}\theta_{2}+(\theta_{1}-\theta_{2}){\left(g\lambda +\phi \right)}^{2}\right)}^{2}/(288\beta \theta_{1}^{2}\theta_{2}^{2})$;
(F, E)	$c_{l}^{}<c_{e}<c_{h}^{}$	$e_{1}^{*}=g\lambda /(2\theta_{1})$; $p_{f1}^{FE*}=(g^{2}\lambda^{2}-g\lambda \theta_{1}+3\beta \theta_{1}+c_{e}\theta_{1}+\delta \theta_{1})/(3\theta_{1})$; $p_{e2}^{FE*}=(-g^{2}\lambda^{2}+4g\lambda \theta_{1}+12\beta \theta_{1}+8c_{e}\theta_{1}-4\delta \theta_{1})/(12\theta_{1})$; $\pi_{1}^{FE*}={\left((-4g\lambda +12\beta +4c+4\delta )\theta_{1}+g^{2}\lambda^{2}\right)}^{2}/(288\beta \theta_{1}^{2})$;$\pi_{2}^{FE*}={\left((4g\lambda +12\beta -4c-4\delta )\theta_{1}-g^{2}\lambda^{2}\right)}^{2}/(288\beta \theta_{1}^{2})$;
(E, E)	$c_{e}<c_{l}^{}$	$p_{e1}^{EE*}=\beta +c_{e};p_{e2}^{EE*}=\beta +c_{e}$; $\pi_{1}^{EE*}=\beta /2;\pi_{2}^{EE*}=\beta /2$.

Note:$c_{l}^{}=-(g^{2}\lambda^{2}-4g\lambda \theta_{1}+4\delta \theta_{1})/(4\theta_{1})$, $c_{h}^{}=-(g^{2}\lambda^{2}-4g\lambda \theta_{2}+4\delta \theta_{2})/(4\theta_{2})$. Suppose ${\underline{c}}^{}<c_{e}<{\bar{c}}^{}$ and $\theta_{2}-\theta_{1}<12\beta \theta_{1}\theta_{2}/(g^{2}\lambda^{2})$, where ${\underline{c}}^{}=-(g^{2}\lambda^{2}-4g\lambda \theta_{2}+12\beta \theta_{2}+4\delta \theta_{2})/(4\theta_{2})$ and ${\bar{c}}^{}=-(g^{2}\lambda^{2}-4g\lambda \theta_{1}-12\beta \theta_{1}+4\delta \theta_{1})/(4\theta_{1})$ (Q.E.D, Latin abbreviation “quod erat demonstrandum”, says it has been proven).

**Proof** **of** **Corollary** **1** Based on equilibrium outcomes in Table A4, we can get

(A7) $$\partial p_{f1}^{FF*}/\partial \theta_{1}=\partial p_{f1}^{FE*}/\partial \theta_{1}=-g^{2}\lambda^{2}/(3\theta_{1}^{2})<0,\;\partial p_{f2}^{FF*}/\partial \theta_{2}=-g^{2}\lambda^{2}/(3\theta_{2}^{2})<0,\;\;\partial c_{l}/\partial \theta_{1}=g^{2}\lambda^{2}/(4\theta_{1}^{2})>0,\;\partial c_{l}/\partial \theta_{2}=0,\;\partial c_{h}/\partial \theta_{1}=0,$$

and

(A8) $$\partial c_{h}/\partial \theta_{2}=g^{2}\lambda^{2}/(4\theta_{2}^{2})>0.$$

In addition, we get that

(A9) $$\partial p_{f1}^{FF*}/\partial \lambda =g^{2}\lambda (4\theta_{2}-\theta_{1})/(6\theta_{1}\theta_{2})>0\;\mathrm{and}\;\partial p_{f1}^{FE*}/\partial \lambda =\partial p_{e2}^{FE*}/\partial \lambda =g(2g\lambda -\theta_{1})/(3\theta_{1})>0.$$

Solving $\partial p_{f2}^{FF*}/\partial \lambda >0$, we can get $g^{2}\lambda (4\theta_{1}-\theta_{2})/(6\theta_{1}\theta_{2})>0$, i.e., $4\theta_{1}>\theta_{2}$. Corollary 1 is proven (Q.E.D). □

**Proof** **of** **Corollary** **2** Based on the equilibrium outcomes in Table A4, we can get

(A10) $$\begin{array}{l} \partial \pi_{1}^{FE*}/\partial c_{e}=(g^{2}\lambda^{2}+4\theta_{1}(-g\lambda +3\beta +c_{e}+\delta ))/(36\beta \theta_{1})>0\\ \partial \pi_{2}^{FE*}/\partial c_{e}=(g^{2}\lambda^{2}+4\theta_{1}(-g\lambda -3\beta +c_{e}+\delta ))/(36\beta \theta_{1})<0,\;\partial \pi_{1}^{EE*}/\partial c_{e}=\partial \pi_{2}^{EE*}/\partial c_{e}=0 \end{array}$$

The Corollary 2 is proven (Q.E.D). □

**Proof** **of** **Corollary** **3** Based on the equilibrium conditions in the proof of Propositions 1 and 2, we get

(A11) $$c_{l}^{s}-c_{l}=-\phi (2g\lambda +\phi -4\theta_{1})/(4\theta_{1})>0,c_{h}^{s}-c_{h}=-\phi (2g\lambda +\phi -4\theta_{2})/(4\theta_{2})>0.$$

According to the equilibrium outcomes with and without a consumer subsidy (in Table A2 and Table A4, respectively), we deduce that $e_{1}^{s*}-e_{1}^{*}=\phi /(2\theta_{1})>0,e_{2}^{s*}-e_{2}^{*}=\phi /(2\theta_{2})>0$, $p_{f1}^{FFs*}-p_{f1}^{FF*}=\phi (4\theta_{2}-\theta_{1})(2g\lambda +\phi )/(12\theta_{1}\theta_{2})>0$, $p_{e2}^{FEs*}-p_{e2}^{FE*}=\phi (4\theta_{1}-2g\lambda -\phi )/(12\theta_{1})>0$ (because of the assumption $2\theta_{1}>2g\lambda +\phi$),

(A12) $$p_{e1}^{EEs*}=p_{e1}^{EE*}=p_{e2}^{EEs*}=p_{e2}^{EE*}=\beta +c_{e}$$

Besides, due to $p_{f2}^{FFs*}-p_{f2}^{FF*}=\phi (4\theta_{1}-\theta_{2})(2g\lambda +\phi )/(12\theta_{1}\theta_{2})$, we can easily get that $p_{f2}^{FFs*}>p_{f2}^{FF*}$ if $\theta_{2}<4\theta_{1}$ holds, verse vice. Similarly, owing to $p_{f1}^{FEs*}-p_{f1}^{FE*}=\phi (2g\lambda +\phi -\theta_{1})/(3\theta_{1})$, we can get that $p_{f1}^{FEs*}>p_{f1}^{FE*}$ if $\theta_{1}<2g\lambda +\phi$ holds, and $p_{f1}^{FEs*}<p_{f1}^{FE*}$ if $\theta_{1}>2g\lambda +\phi$ holds. The Corollary 3 is proven (Q.E.D). □

**Proof** **of** **Proposition** **3** The total carbon emissions generated by the two firms are given in Table 3. By taking the equilibrium results $e_{i}^{*}$ and $p_{ki}^{*}$ into $d_{ki}^{}$ and $CE^{mns*},i\in \{1,2\},m,n\in \{E,F\},k\in \{e,f\}$, it is easy to prove that

(A13) $$\begin{array}{l} CE^{FFs*}-CE^{FF*}=g(1-e_{1}^{s*})d_{f1}^{s*}+g(1-e_{2}^{s*})d_{f2}^{s*}-g(1-e_{1}^{*})d_{f1}^{*}-g(1-e_{2}^{*})d_{f2}^{*}=-g\lambda (3g\lambda (g\lambda +\phi )\\ (\theta_{1}-\theta_{2}{)}^{2}+\phi^{2}{\left(\theta_{1}-\theta_{2}\right)}^{2}+12\beta \theta_{1}\theta_{2}(\theta_{1}+\theta_{2}))/(48\beta \theta_{1}^{2}\theta_{2}^{2})<0. \end{array}$$

The Proposition 3 is proven (Q.E.D). □

**Proof** **of** **Proposition** **4** Based on Table A2, we can calculate that

(A14) $$\begin{array}{l} \partial \pi_{1}^{FFs*}/\partial \phi =({\left(g\lambda +\phi \right)}^{3}{\left(\theta_{2}-\theta_{1}\right)}^{2}+12\beta \theta_{1}\theta_{2}(g\lambda +\phi )(\theta_{2}-\theta_{1}))/(72\beta \theta_{1}^{2}\theta_{2}^{2})>0,\\ \partial \pi_{2}^{FFs*}/\partial \phi =(g\lambda +\phi )(\theta_{1}-\theta_{2})({\left(g\lambda +\phi \right)}^{2}+12\beta \theta_{1}\theta_{2})/(72\beta \theta_{1}^{2}\theta_{2}^{2})<0,\\ \partial \prod_{}^{FFs*}/\partial \phi ={\left(\theta_{2}-\theta_{1}\right)}^{2}{\left(g\lambda +\phi \right)}^{3}/(36\beta \theta_{1}^{2}\theta_{2}^{2})>0,\\ \partial \pi_{1}^{FEs*}/\partial \phi =({\left(g\lambda +\phi \right)}^{2}+\theta_{1}(-4g\lambda +12\beta +4c_{e}+4\delta -4\phi ))(g\lambda +\phi -2\theta_{1})/(72\beta \theta_{1}^{2})<0,\\ \partial \pi_{2}^{FEs*}/\partial \phi =({\left(g\lambda +\phi \right)}^{2}+\theta_{1}(-4g\lambda -12\beta +4c_{e}+4\delta -4\phi ))(g\lambda +\phi -2\theta_{1})/(72\beta \theta_{1}^{2})>0,\\ \partial \pi_{1}^{EEs*}/\partial \phi =0,\;\mathrm{and}\;\partial \pi_{2}^{EEs*}/\partial \phi =0. \end{array}$$

Besides, to ensure $\partial \prod_{}^{FEs*}/\partial \phi <0$ where $\partial \prod_{}^{FEs*}/\partial \phi =({\left(g\lambda +\phi \right)}^{2}+\theta_{1}(-4g\lambda +4c_{e}+4\delta -4\phi ))(g\lambda +$$\phi -2\theta_{1})/(36\beta \theta_{1}^{2})$, we can get the condition 0<ϕ<Δϕ, where $\Delta \phi =2\theta_{1}-g\lambda -2\sqrt{\theta_{1}^{2}-c_{e}\theta_{1}-\delta \theta_{1}}$. The Proposition 4 is proven (Q.E.D). □

**Proof** **of** **Proposition** **5** By purchasing vehicles produced by firm 1 and firm 2, the consumer surplus function is given in Table 4. Specifically, $CS^{FF*}=\int_{0}^{d_{f1}}(v_{f1})dx+\int_{d_{f1}}^{1}(v_{f2})dx$ and $CS^{FE*}=\int_{0}^{d_{f1}}(v_{f1})dx+\int_{d_{f1}}^{1}(v_{e2})dx$. By taking $e_{i}^{*}/e_{i}^{s*}$ and $p_{ki}^{*}/p_{ki}^{s*}$ into the Equations (A1)–(A4), and then taking $v_{k1}$ and $v_{k2}$ into $CS^{mn}$, we can get

(A15) $$\begin{array}{l} \partial CS^{FFs*}/\partial \phi =(g\lambda \theta_{1}-2g\lambda \theta_{2}+\phi \theta_{1}-\phi \theta_{2})(g\lambda \theta_{1}+\phi \theta_{1}-\phi \theta_{2})+\theta_{2}(g^{2}\lambda^{2}\theta_{2}+36\beta \theta_{1}^{2}+36\beta \theta_{1}\theta_{2})\;\mathrm{and}\\ \partial CS^{FEs*}/\partial \phi =((g\lambda +\phi -2\theta_{1})(4\theta_{1}(\delta +c_{e})+{\left(g\lambda +\phi \right)}^{2}-4(g\lambda +\phi )\theta_{1})+36\beta (g\lambda +\phi +2\theta_{1}))/(144\beta \theta_{1}^{2}). \end{array}$$

Solving $\partial CS^{FEs*}/\partial \phi <0$, we find conditions 0<ϕ<Δϕ and δ>Δδ should be both satisfied, where $\Delta \phi =2\theta_{1}-g\lambda -2\sqrt{\theta_{1}^{2}-c_{e}\theta_{1}-\delta \theta_{1}}$ and $\Delta \delta =9\beta (2\theta_{1}+g\lambda +\phi )/(2\theta_{1}-g\lambda -\phi )-(4c_{e}\theta_{1}+{\left(g\lambda +\phi \right)}^{2}-$$4(g\lambda +\phi )\theta_{1})/(4\theta_{1})$. The Proposition 5 is proven (Q.E.D). □
